# Supplementary material for: Safety and effectiveness of monovalent COVID-19 mRNA vaccination and risk factors for hospitalisation caused by the omicron variant in 0.8 million adolescents: A nationwide cohort study in Sweden
Source: PLoS Med. 2023 Feb 21;20(2):e1004127. doi: 10.1371/journal.pmed.1004127 (PMC9990916; doi:10.1371/journal.pmed.1004127)
Supplement: S1 Table — Data publicly available at the Public Health Agency of Sweden (https://www.folkhalsomyndigheten.se/smittskydd-beredskap/utbrott/aktuellautbrott/covid-19/statistik-och-analyser/sars-cov-2-virusvarianter-av-sarskild-betydelse/). (PDF) [file pmed.1004127.s002.pdf]

Supplementary Appendix to:

Safety and effectiveness of monovalent COVID-19 mRNA vaccination and risk factors for hospitalisation caused by the omicron variant in 0.8 million adolescents: A nationwide cohort study in Sweden

Prof. Peter Nordström, PhD[1,2], Marcel Ballin, MSc[2], Anna Nordström, PhD[3,4]

Affiliations

[1]Department of Public Health and Caring Sciences, Clinical Geriatrics, Uppsala University, Uppsala, Sweden
[2]Department of Community Medicine and Rehabilitation, Unit of Geriatric Medicine, Umeå University, Umeå, Sweden (Marcel Ballin)
[3]Department of Public Health and Clinical Medicine, Section of Sustainable Health, Umeå University, Umeå, Sweden (Anna Nordström)
[4]School of Sport Sciences, UiT the Arctic University of Norway, Tromsø, Norway (Anna Nordström)

Contents

Supplementary Table 1. Type of SARS-CoV-2 genotypes based on whole genome sequencing in Sweden during the follow-up period of the present study (week 1-22, 2022). Data publicly available at the Public Health Agency of Sweden (https://www.folkhalsomyndigheten.se/smittskydd-beredskap/utbrott/aktuella-utbrott/covid-19/statistik-och-analyser/sars-cov-2-virusvarianter-av-sarskild-betydelse/) 2

Supplementary Table 1. Type of SARS-CoV-2 genotypes based on whole genome sequencing in Sweden during the follow-up period of the present study (week 1-22, 2022). Data publicly available at the Public Health Agency of Sweden (https://www.folkhalsomyndigheten.se/smittskydd-beredskap/utbrott/aktuella-utbrott/covid-19/statistik-och-analyser/sars-cov-2-virusvarianter-av-sarskild-betydelse/)

	Type of SARS-CoV-2 variant							
**Week number , 2022**	**Delta (B.1.617.2) Number of cases**	**Omicron (B.1.1.529) Number of cases**	**Omicron (BA.1) Number of cases**	**Omicron (BA.2) Number of cases**	**Omicron (BA.4) Number of cases**	**Omicron (BA.5) Number of cases**	**Total number of whole genome sequenced**	**Proportion whole genome sequenced of all confirmed cases (%)**
1	365	233	3 158	474			4 353	3
2	173	320	3 468	905			4 999	3
3	54	323	2 827	1 458			4 876	2
4	21	132	2 447	2 061			4 876	2
5	5	229	2 223	3 237			5 942	3
6	1	258	1 328	3 113			4 864	8
7	0	238	865	2 612			3 916	18
8	0	168	563	2 908			3 758	21
9	0	105	369	2 972			3 536	28
10	0	136	215	2 899			3 331	33
11	0	111	116	2 405			2 684	32
12	0	132	45	2 061			2 248	32
13	0	139	21	1 548			1 723	35
14	0	115	16	1 260			1 416	35
15	0	16	11	1 077	0	5	1 159	39
16	0	1	4	917	0	3	954	40
17	0	1	2	727	2	1	741	36
18	0	0	4	677	5	7	702	41
19	0	0	2	742	11	11	781	54
20	0	1	1	658	21	26	727	53
21	0	1	0	564	27	58	659	56
22	0	4	0	518	30	118	675	55
