# Supplementary material for: Safety and effectiveness of monovalent COVID-19 mRNA vaccination and risk factors for hospitalisation caused by the omicron variant in 0.8 million adolescents: A nationwide cohort study in Sweden
Source: PLoS Med. 2023 Feb 21;20(2):e1004127. doi: 10.1371/journal.pmed.1004127 (PMC9990916; doi:10.1371/journal.pmed.1004127)
Supplement: Attachment — Submitted filename: Authors responses 221225.docx [file pmed.1004127.s003.docx]

Requests from the editors:

GENERAL

1. Please respond to all editor and reviewer comments detailed below, in full

- **Authors’ response:** Done!

2. Please ensure that the study is reported according to the STROBE guideline, and include the completed STROBE checklist as Supporting Information.  Please add the following statement, or similar, to the Methods: "This study is reported as per the Strengthening the Reporting of Observational Studies in Epidemiology (STROBE) guideline (S1 Checklist)." The STROBE guideline can be found here: http://www.equator-network.org/reporting-guidelines/strobe/. When completing the checklist, please use section and paragraph numbers, rather than page numbers.

- **Authors’ response:** Done**.**

DATA AVAILABILITY STATEMENT

3. Thank you for including a Data Availability Statement (DAS) which requires revision. For each data source used in your study:

 a) If the data are freely or publicly available, note this and state the location of the data: within the paper, in Supporting Information files, or in a public repository (include the DOI or accession number).
 b) If the data are owned by a third party but freely available upon request, please note this and state the owner of the data set and contact information for data requests (web or email address). Note that a study author cannot be the contact person for the data.
c) If the data are not freely available, please describe briefly the ethical, legal, or contractual restriction that prevents you from sharing it. Please also include an appropriate contact (web or email address) for inquiries (again, this cannot be a study author).

- **Authors’ response:** We have revised the data availability statement as follows:

  *“The data underlying the findings of the present study were used under ethical approval and are publicly unavailable according to regulations under Swedish law. Researchers who are interested in obtaining the data can contact Statistics Sweden via mikrodata@scb.se (https://www.scb.se/en/services/ordering-data-and-statistics/ordering-microdata/), National Board of Health and Welfare via registerservice@socialstyrelsen.se (https://bestalladata.socialstyrelsen.se), and Public Health Agency of Sweden via info@folkhalsomyndigheten.se (https://www.folkhalsomyndigheten.se/the-public-health-agency-of-sweden/)”*

COMMENTS FROM THE ACADEMIC EDITOR

1. It would be of value, if the authors explain why they prefer to select matched controls and thereafter adjust for the matching variables - instead of running the full cohort with adjustments. Less risk of introducing bias in the latter alternative. Further, I would like to underline reviewer #3’s request to, if possible, include more potential confounders from available registers.

- **Authors’ response: This is an insightful question, although probably due to a misunderstanding of the rather complex set up and many analyses, which we are happy to clarify. To each vaccinated individual, Statistics Sweden matched one control. This total cohort was then delivered to us. From this cohort, we performed several analyses, and in each analysis we included all individuals that was possible. To give one example, in the main VE analysis, we included all individuals vaccinated with at least two doses and all never-vaccinated individuals, excluding those with a prior documented SARS-CoV-2 infection at baseline. Given that some individuals were excluded, this resulted in slightly different baseline characteristics in the different groups that we compared as presented in Table 1. Therefore, we adjusted also for variables that Statistics Sweden initially matched for. With respect to the question to include more potential confounders in the analysis. As presented, we had access to a huge number of potential confounders (diagnoses at baseline). However, given that this cohort consisted of healthy adolescents, most of the diagnoses were rare, and the diagnoses were also rather well balanced between vaccinated individuals and controls (please see Table 1). Thus, adjusting for these diagnoses (binary variables) in the analyses would not be advisable and would likely result in unstable regression estimates.**

ABSTRACT

2. Abstract Background: Please ensure that the final sentence clearly states the study question.

- **Authors’ response:** We have now added a clear statement of the study question to the Background section in the Abstract:

*“The aim of the present study was therefore to examine the safety and effectiveness of monovalent COVID-19 mRNA vaccination against COVID-19 hospitalisation, and risk factors for COVID-19 hospitalisation in adolescents*”

3. Line 44: Please include a summary of the adverse events referred to.

- **Authors’ response:** We have now revised this paragraph to read as follows:

*“The safety analysis showed that COVID-19 mRNA vaccination was not associated with an increased risk of hospitalisation for any cause”*

4. Abstract methods and findings: Line 49-50: when reporting statistical information suggest placing a colon after OR instead of a comma, placing CIs within square parentheses and using lower case p, as follows: (OR: 12.0, 95% CI [6.4, 22.6], p<0.001) to improve accessibility to the reader.  Please check and amend throughout the manscript. The use of commas between upper and lower limits adds clarity over the use of hyphens where negative values are reported.

- **Authors’ response:** Good point. We have corrected this throughout the manuscript.

5. Please include any important dependent variables that are adjusted for in the analyses.

- **Authors’ response: Good point, this has been added.**

6. In the last sentence of the Abstract Methods and Findings section, please describe the main limitation(s) of the study's methodology.

- **Authors’ response:** We have now added the following limitations to this section:

*“Limitations of this study include the observational design and the possibility of unmeasured confounding.”*

7. Abstract conclusions: Line 60: suggest general population of adolescents perhaps? Or something similar

- **Authors’ response:** Good suggestion. We revised to the following:

*“However, COVID-19 hospitalisation in the general population of adolescents was extremely rare…”*

AUTHOR SUMMARY

8. Thank you for including an author summary. Please change the first sub-heading to “Why was this study done?”

- **Authors’ response:** Corrected.

9. Line 80: starting at “In addition...” suggest this is a separate bullet point.

- **Authors’ response:** Agree, and corrected.

10. Line 96 onwards: suggest “Clear risk factors for COVID-19 hospitalisation included infections and different developmental disorders, resulting in over a tenfold increased risk of hospitalisation.  Vaccine effectiveness among these individuals was similar as in the rest of the cohort.” – I think it might help to elaborate a little on the infections that you mention – what kind? Perhaps would be helpful to broaden the definition in the abstract also.

- **Authors’ response:** Good point! We have now elaborated on this here in the Author Summary and also added it in the Abstract

  *“There were certain strong risk factors for COVID-19 hospitalisation, including previous infections, (such as bacterial infection, tonsillitis, and pneumonia), and different development disorders, which increased the risk of COVID-19 hospitalisation more than tenfold.”*

11. Line 103: as above suggest “general population of adolescents” or something similar.

- **Authors’ response:** Corrected.

12. Suggest removing the word “Therefore” and beginning with “Administration…” as a separate bulleted point.

- **Authors’ response:** Corrected.

13. Line 105: suggest an individually bulleted point for this statement, which is of significant importance

- **Authors’ response:** Corrected.

METHODS AND RESULTS

14. Did your study have a prospective protocol or analysis plan? Please state this (either way) early in the Methods section.
    a) If a prospective analysis plan (from your funding proposal, IRB or other ethics committee submission, study protocol, or other planning document written before analyzing the data) was used in designing the study, please include the relevant prospectively written document with your revised manuscript as a Supporting Information file to be published alongside your study, and cite it in the Methods section. A legend for this file should be included at the end of your manuscript. 
    b) If no such document exists, please make sure that the Methods section transparently describes when analyses were planned, and when/why any data-driven changes to analyses took place.  
    c) In either case, changes in the analysis-- including those made in response to peer review comments-- should be identified as such in the Methods section of the paper, with rationale.

- **Authors’ response:** The present study did not have a formal prospective protocol or analysis plan. This has been added to the manuscript with an explanation (page 7, line 155).

15. For all observational studies, we ask that authors ensure the following has been clearly indicated in the manuscript text:

     (1) the specific hypotheses you intended to test,

     (2) the analytical methods by which you planned to test them,

     (3) the analyses you actually performed, and

     (4) when reported analyses differ from those that were planned, transparent explanations for
 differences that affect the reliability of the study's results. If a reported analysis was
 performed based on an interesting but unanticipated pattern in the data, please be clear that
 the analysis was data-driven.

**Authors’ response: Ok, done**

16. Line 194: “….as per when the guidelines for testing in Sweden had changed.” Suggest instead “…in-line with the changes made to testing guidelines in Sweden” or something similar.

- **Authors’ response:** We have now corrected to:

  *“…in-line with the changes made to testing guidelines in Sweden.”*

17. Please remove “role of the funding source” from the end of the methods section (line 244)

- **Authors’ response:** Done.

18. Line 254 onwards: where you report p-values please also report 95% CIs.

- **Authors’ response:** We have added this throughout where possible. For example, when the statistical method to compare groups is a t-test, there is no 95% CIs.

19. Line 271: as for the abstract please revise the presentation of statistical information 75% (95% CI [54-86], p<0.001). Please revise throughout to ensure consistency and clarity

- **Authors’ response:** We have now corrected this throughout the manuscript.

20. Line 306: (VE, 4%, 95% CI, 2-6, 307 P<0.001) perhaps a semicolon following VE? And parentheses and commas for CIs as previously detailed

- **Authors’ response:** Agree, and corrected throughout.

21. Line 315: “…with no detectable VE (VE, -6%, 95% CI, -51-119, P=0.88)” is confusing to me, please check and revise accordingly. Please also see reviewer #3 comments.

- **Authors’ response:** The use of influenza as a negative control outcome was included to explore potential unmeasured confounding. Because COVID-19 vaccination is not thought to be causally related to a lower risk of hospitalization due to influenza, any detected vaccine effectiveness in favor of such an association would provide evidence that there might be confounding in our study. However, as shown, two doses of COVID-19 vaccine were not associated with reduce risk of hospitalization due to influenza, as shown by the fact that we did not detect any vaccine effectiveness. With respect to reviewer #3’s comment, the reason why the upper end of the 95% CI exceeds 100 is because the VE is based on the OR and the 95% CI surrounding it. VE = 1-OR x 100. We have now revised this section to clarify and improve readability:

  *“The results showed that individuals vaccinated with two doses of COVID-19 vaccine did not experience a lower risk of hospitalisation due to influenza as compared to never vaccinated individuals (VE; -10%, 95% CI [-133, 48], P=0.81), thus indicating an absence of important unmeasured confounding.”*

22. We agree with the academic editor and the reviewer #3 that adjustment for additional covariates would be beneficial.  Please include if possible, if not, please clearly state the reason why.

- **Authors’ response: Please see the answer to the first point of the Academic editor.**

FIGURES

23. Line 521 details “Figure legends” but I cannot see any legends in my version of the manuscript or that nay are required, please remove.

- **Authors’ response:** Removed.

24. Figure 1 – please ensure that all numerical values presented are identical to those in the manuscript text

- **Authors’ response:** Done

TABLES

25. Table 2: Please also present the unadjusted analyses for comparison.  As for the adjusted analyses please report 95% CIs and p-values.

- **Authors’ response:** Done

26. Table 3 and 4: please also report p-values where you report 95% CIs for adjusted and unadjusted analyses

- **Authors’ response:** Done

DISCUSSION

27. Please remove the sub-heading “conclusions “ such that the discussion reads as single piece of continuous prose ending in the one paragraph conclusion.

- **Authors’ response:** Done.

28. Please remove declaration statements from the end of the discussion (line 405, 408, 411) and include only in the submission form when you re-submit your manuscript.

- **Authors’ response:** Done.

REFERENCES

29. In text reference call outs should be placed within square brackets preceding punctuation as follows: Line 128 – “alleviated their restrictions [1].” As opposed to “alleviated their restrictions.[1]”
30. Please also remove spaces between citations where more than one study is cited i.e. [1,2,3,4] as opposed to [1, 2, 3, 4]
31. In the reference list please include up to but no more than 6 author names followed by et al where more than 6 authors contribute to a study
32. Please use the "Vancouver" style for reference formatting, and see our website for other reference guidelines https://journals.plos.org/plosmedicine/s/submission-guidelines#loc-references
33. Journal name abbreviations should be those found in the National Center for Biotechnology Information (NCBI) databases.

- **Authors’ response:** Corrected.

Comments from the reviewers:

Reviewer #1: This paper describes the vaccine effectiveness and safety of mRNA COVID-19 vaccines among adolescents in Sweden during Omicron predominance, which is an important and timely topic. VE estimates against hospitalization for adolescents during Omicron predominance are needed, and thus the paper adds important evidence.  The paper will be improved by additional explanation of methods and, if possible, inclusion of myocarditis as a specific safety outcome. My specific comments are below:

Specific comments:

1. The authors need to further explain the infection eligibility in the cohort. I don't understand the purpose of this eligibility and matching criteria nor if it was used for an analysis. The authors need to better explain that. This is described as a cohort study, in which case there should not be any selection based on the outcome (SARS-COV-2 infection or hospitalization).

- **Authors’ response:** We understand that the description of the study cohort is complex. We have simplified the description and removed the controls that was matched to individuals with a previous infection. This means that we included all individuals with at least one dose of vaccine and matched controls. In this cohort we could track all SARS-CoV-2 infections before and after baseline since we also had access to data on all documented infections in Sweden. Figure 1, the tables and the text has been changed accordingly.

2. The overall conclusion that adolescents may not need COVID-19 vaccination is based on low absolute risk of hospitalization, but this doesn't account for long-COVID or risk of MIS-C, which should be discussed. Additionally, the VE was 75% for hospitalization, meaning that there was a 75% risk reduction in hospitalization for adolescents after 2 doses of vaccination.

- **Authors’ response:** The reviewer is correct in that COVID-19 vaccination was associated with 75% relative risk reduction of COVID-19 hospitalization, which is also specifically stated in the paper. However, from a risk/benefit perspective and vaccination policy it should arguably be put into context with the absolute risk, which was extremely low, as correctly pointed out also by the reviewer. With respect to long- COVID or MIS-C, we looked at the number of cases, finding that only 3 and 6 individuals, respectively, were diagnosed in specialized open health care during the total follow up time in the total cohort. However, we prefer not to include these data in the present study since these diagnoses may also be set in primary health care which is not covered by the Swedish National Patient Register.

3. Also, the outcome of all-cause hospitalization for safety is important. Can the authors potentially also examine myocarditis/pericarditis for a safety outcome? If they cannot, they should mention myocarditis/pericarditis in the discussion.

- **Authors’ response:** This comment may perhaps be due to misunderstanding. We have indeed examined myocarditis and pericarditis as specific safety outcomes. These results are presented in Table 2, showing no increased risk of these outcomes in vaccinated individuals.

4. Given that bivalent mRNA vaccines are now in use in multiple countries, the authors need to clearly specify in the title and abstract that these data reflect monovalent mRNA vaccines.

- **Authors’ response:** Good point. We agree and have corrected accordingly.

5. Abstract: The sentence regarding vaccine safety should specify that it is all-cause hospitalizations and that vaccinated in that sentence means ≥1 mRNA COVID-19 vaccine dose (which is different from the VE definition).

- **Authors’ response:** We are not sure exactly which sentence the reviewer means (as there was no lines specified), but we have now ensured that it is very clear that the safety analysis considers at least 1 dose of vaccine, and the VE analysis 2 doses. We also clarified which outcomes were considered in the safety analysis. Please see the revised Abstract.

6. Abstract: Specify what previous infections are risk factors for COVID-19 hospitalizations. Do you mean prior COVID-19 infection as that would be counterintuitive? If this means prior infections, please specify of what and how this is measured.

- **Authors’ response:** Thanks for letting us clarify. We have now revised and specified in the Abstract as follows:

*“Strong risk factors for COVID-19 hospitalisation included previous infections (bacterial infection, tonsillitis, and pneumonia) (odds ratio [OR]: 14.3, 95% CI [7.7, 26.6], P<0.001), and cerebral palsy/development disorders (OR: 12.7, 95% CI [6.8, 23.8], P<0.001)”*

7. Abstract conclusion regarding safety states "COVID-19 mRNA vaccination was not associated with an increased risk of any serious adverse event in adolescents" but only data on SAEs associated with hospitalization were presented in the abstract. The conclusion should reflect the data presented.

- **Authors’ response:** We have revised and hopefully improved the Abstract, including the conclusion section. Please see the following:

“*In this nationwide study of Swedish adolescents, monovalent COVID-19 mRNA vaccination was not associated with an increased risk of serious adverse events resulting in hospitalisation. Vaccination with two doses was associated with a lower risk of COVID-19 hospitalisation during an omicron predominant period, also among those with certain predisposing conditions who should be prioritized for vaccination. However, COVID-19 hospitalisation in the general population of adolescents was extremely rare, and additional doses in this population may not be warranted at this stage.”*

8. Summary: regarding this conclusion: "In contrast, individuals with a high risk for severe COVID-19 should be prioritised for vaccination.", it might be helpful to state here whether these individuals with a high risk for severe COVID-19 should be prioritized for primary series, additional doses, or both (likely both).

- **Authors’ response:** Good point. Please see the revised conclusion section as above.

9. The introduction should include a summary of Sweden's COVID-19 vaccine recommendations for adolescents, so that non-Swedish readers can interpret these data in the context of Sweden's recommendations.

- **Authors’ response:** We understand the reviewer’s point, but this is not feasibly. This is because these recommendations have changed numerous times, and recommendations are also different for certain groups that have been considered at higher risk at different time points during the pandemic. We suggest that the results are of higher interest with respect to current recommendations during the Omicron era in different countries.

10. Introduction: Lines 134-135: This sentence implies that the data are inconsistent with myocarditis after COVID-19 vaccine. At this point, the data are fairly clear that there is a rare risk of myocarditis/pericarditis after COVID-19 vaccines (https://www.cdc.gov/vaccines/covid-19/clinical-considerations/myocarditis.html), and the risk is highest in adolescent males (half the population in this study). This sentence should be amended to clarify this point.

- **Authors’ response:** We have now revised this sentence and removed the part suggesting inconsistency.

11. Introduction: Lines 137-139: Please specify the variant period for this 80% VE against hospitalization from prior studies.

- **Authors’ response:** Thanks, we forgot to specify this. We have now added the following:

*“…during the omicron era”*

12. Methods: Lines 164-165: I found this section a bit hard to follow. Were vaccinated adolescents matched to unvaccinated adolescents regardless of infection status? Similarly were adolescents with infection matched to those without regardless of vaccination status? Can you clarify?

- **Authors’ response:** We understand that the matching process is quite complex, but reviewer is correct. Thus, Statistics Sweden matched vaccinated individuals to individuals not vaccinated, regardless of infection status, and infected individuals were matched to never infected individuals, regardless of vaccination status. However, for the present version of the manuscript, we excluded controls that was matched by Statistics Sweden to individuals with infection, to avoid bias. This has hopefully simplified and improved the description of the cohort selected for the present study.

13. Lines 244-245: Role of the funding source: This might be better stated as "no external funding was used for this study," as presumably the authors did this as part of their employment (meaning their time was funded by their employers) and the data likely is funded by Sweden's public health system.

- **Authors’ response:** We have removed the section regarding role of funding as per the Editors’ request, as this is to be stated in the submission system only.

14. Figure 1:

a) It would be helpful to specifically state how many adolescents were excluded with known prior SARS-CoV-2 infection.

- **Authors’ response:** We assume this question concerns individuals used in the VE analysis, and this information can be found in Table 1 for individuals given at least one dose of vaccine and for the controls.

b) It would be also helpful in the paper to specify what the hospitalization rate was among adolescents with any known prior SARS-CoV-2 infection is, as these adolescents would presumably be at lower risk of hospitalization. Given that in some countries (such as the US), the majority of people in this age group now have had prior COVID-19 infection, this question is of direct public health relevance.

- **Authors’ response:** We are not sure about this question. However, it is clear from Table 1 that in Sweden less than 15% of adolescents had a documented previous SARS-CoV-2 infection at baseline for the safety analysis. These individuals were excluded from the VE analysis. In the VE analysis, only 47 individuals were hospitalized due to COVID-19, it seems therefore unlikely that previous infections would have any major impact on later hospitalizations, especially since the majority of individuals who contracted SARS-CoV-2 did so during the omicron era.

15. Table 1: It is unclear what the previous ICD-10 diagnoses are from: any previous diagnosis listed in their medical records? Or it is only from a hospitalization? Is this lifetime or over a certain time frame? All that needs to be specified.

- **Authors’ response:** In the final paragraph of the sub-section Outcomes in the Methods section, as well as in the footnote to Table 1, we have described the meaning of the previous diagnoses in the present study:

  “Data on all diagnoses used in this study were obtained from the National Patient Register[15] using 10^th^ revision International Classification of Disease codes, starting from 1 January 2016 earliest until 5 June 2022 latest”

  “All previous diagnoses were main diagnoses set during inpatient hospital stay, with the exception of the combined diagnoses where also open specialized health care was used”

  However, we agree that the information on the timeframe was missing from the footnote in Table 1, and have therefore revised it accordingly.

*“All diagnoses were main diagnoses set during inpatient hospital stay from 1 January 2016 earliest until 5 June 2022 latest as obtained from the National Patient Register, with the exception of the diagnoses listed under “combined diagnoses” where also diagnoses set during secondary outpatient care was used.”*

16. Results lines 270-275: Please include a median and IQR for times (days if possible) since vaccination. Given that we know that mRNA VE wanes over time in general, but may be more preserved for more severe outcomes, particularly among younger people (like adolescents), any additional data that can be included to allow us to understand whether these data had any suggestion of waning would be very helpful.

**Authors’ response:** Good suggestion. However, to directly present follow up times would not be correct since we only counted outcomes starting from 1^st^ January 2022, to ensure that all cases were related to the Omicron variant. In addition, we already present results suggesting waning VE, both with respect to COVID-19 hospitalizations and for the secondary outcome SARS-CoV-2 infection of any severity, where the VE becomes lower as the time since vaccination increases. Please see the result section, Table 3 and 4.

17. Results lines 276-277: Please include the percent of adolescents with 3 doses who were hospitalized (i.e. 12/41,225 = 0.029%). This percent appears to be higher than for controls (and 2-dose recipients). Is there a reason for this? What was the booster recommendation? How many days had it been since the booster dose?

- **Authors’ response:** We were probably not clear enough in the previous version of the paper. In the cohort used for compared 3 doses vs 2 doses, there were 12 cases in total. Thus (12 / 454769) = 2.6 per 100000 individuals, which is lower than the 7 per 100000 individuals in the cohort comparing 2 doses vs unvaccinated.

18. Results lines 283-285: Among those previously hospitalized for another condition, during what time frame were they hospitalized (over their lifetime versus during a specific timeframe)?

- **Authors’ response:** Please see our response to your previous comment; the timeframe for previous diagnoses was since 1 January 2016 earliest. However, for the specific diagnoses presented in Table 1, we now instead only use diagnoses since 2020 and onwards to avoid bias, given that more individuals among those not vaccinated were not born in Sweden. Thus, if including a longer time frame there is a risk that individuals born outside of Sweden would not have immigrated to Sweden yet, and thus a risk of bias suggesting that these diagnoses would be less common at baseline among individuals not vaccinated.

19. Results 285-289: What kind of infection? More specificity on how you defined these ICD codes included in infection needs to be included. What other buckets of previous diagnoses were examined? That is unclear to me. I presume that this is a marker for being higher risk for hospitalization with infections, but do we know why that is? Are these kids with other underlying conditions, such as asthma, technology dependence, immunosuppression, etc?

- **Authors’ response**: This is an insightful comment. Regarding other previous diagnoses, we studied all previous diagnoses in the 47 individuals hospitalized for COVID-19 in detail without any restrictions. In summary, some of the individuals diagnosed with previous infections were also diagnosed with development disorders. However, apart from this finding there were no clear patterns. This was perhaps expected since COVID-19 hospitalization was very rare. As per the reviewer’s previous comments regarding what is included in previous infections, we have now clarified it at this section as well, just as we have done in the Abstract and Research Summary. Please see the revised part in the Results section:

*“Among the individuals hospitalised due to COVID-19, 15 (32%) had previously been diagnosed with an infection (bacterial infection, tonsillitis, and pneumonia), compared to 22,110 (3.3%) in the rest of the cohort…”*

20. Results: was there overlap between the group previously diagnosed with infection and those with CP or developmental delay?

- **Authors’ response:** Yes! Please see the answer to the comment above.

21. Table 2: How were these 30 diagnostic groups defined? Were they from a list of pre-specified potential COVID risk factors or some other list (like complex chronic conditions of childhood) that has been shown to affect risk of hospitalization or death? That needs to be defined.

- **Authors’ response:** This is described in the Methods section. Please see the following:

*“In the safety analysis, based on all diagnoses set in the cohort during inpatient hospital stay until 5 June 2022, results are presented for all-cause hospitalisation and for 30 different diagnoses. The 30 specific diagnoses were selected based on their incidence and general interest given previous reports of links between certain diagnoses and COVID-19 mRNA vaccination [4, 12, 13]. Only the first diagnosis was evaluated for each individual, and individuals with this diagnosis at baseline were therefore excluded from the prospective analyses.”*

22. Table 3: Please include the first date of vaccine eligibility for these adolescents and if possible, median and IQR for days since vaccination.

**Authors’ response:** We are not sure about the comment related to days since vaccination, since baseline date was the date of vaccination in vaccinated individuals. Furthermore, since the vaccinated individuals represent the total Swedish population in the studied age group, some individuals were vaccinated already early 2021.

To present any data that may be interpreted as follow up time would not be correct, as previously explained, since we only accounted outcomes from 1^st^ January 2022. Thus, individuals with a baseline date earlier than 30^th^ December 2021 could not have a follow-up time for Covid infection of one day.

23. Table 4: Please include the first date of vaccine eligibility for these adolescents (I am guessing it is September 1, 2021) and if possible, median and IQR for days since vaccination.

**Authors’ response**: Please see the answer to the point above.

24. Discussion: Also, what was the infection-induced immunity seroprevalence in Sweden during this time, especially during adolescents? That would helpful add this (or anything known about this) to the discussion to put these results into context.

**Authors’ response:** Please see Table 1 with respect to the number of individuals with a documented previous SARS-CoV-2 infection at baseline.

Reviewer #2: Alex McConnachie, Statistical Review
Nordstrom et al report on a nationwide, retrospective cohort study, looking at the association between COVID-19 mRNA vaccination, and subsequent adverse events and COVID-19 hospitalisations and infections during the omicron wave. This review considers the statistical aspects of the paper. In short, these are very good. The population, exposures, and outcomes are clearly described. The statistical methods are well described and appropriate. A negative control analysis is also included. A couple of minor points:

- **Authors’ response:** Thanks, very happy for this feedback, tried our best!

1. Was the PH assumption checked in the Cox models?

- **Authors’ response:** Yes, and since the associations were time dependent for the outcome of SARS-CoV-2 infection, logistic regression was used in the models to estimate VE. We also presented data showing these time dependent effects in Table 3 and Table 4, although we did not present the information regarding the PH assumption in the Statistics section. This has now been added.

2. Line 278: I find hyphens don't work in confidence intervals where the values can be negative.

- **Authors’ response:** Good point, and this has now been corrected throughout the manuscript and in tables.

Reviewer #3:

1. Very important study. My main objection to this study is that vaccination depends heavily on underlying factors (health seeking behaviour, underlying diseases/comorbidity, perhaps smoking, level of education etc etc), that either a heavily adjusted model or a propensity score model would have been better for this study. Voluntary/"Elective" vaccinations (e.g. influenza) are often linked to "healthy behaviour" (influenza vaccinations typically protect against death all-year round, even when influenza is not around). if that is the case with covid.19 vaccination, then that would lead to a lower risk of hospitalisation for most things, including covid-19 (the so called vaccine efficiency) in this study.

- **Authors’ response:** Very important point and we strongly agree that there is risk of selection bias and residual as well as unmeasured confounding in studies evaluating vaccine effectiveness. However, please note that we used influenza as a negative control, and that COVID-19 vaccination was not associated with this outcome, thus indicating absence of important unmeasured confounding. Please also note that the vaccinated individuals and controls were very similar in the 30 diagnoses at baseline. In addition, it should be noted that VE decreased with time since vaccination, supporting causal effects. Finally, an important finding of the present study is that COVID-19 hospitalization was overall very rare, which questions whether booster doses to the general populations of adolescents should be recommended. This finding is not directly influenced by bias or confounders.

2. Abstract, I am particularly happy to see that the authors present absolute risks/numbers needed to treat. I fully agree that needing to vaccinate >9000 teenagers to save 1 hospital admission for covid-19 (with 0 mortality during the Omicron period), argues against general vaccination in this age group.

- **Authors’ response:** Thank you for recognizing this, and we agree that this is perhaps the most important conclusion of the study, as discussed in the point above.

3. Research summary; what did the researchers do and fin, please clarify that individuals were followed for 6 months. (study period), to say you followed them until June 2022 says rather little.

- **Authors’ response:** Good point. Corrected.

4. I do not understand the statement that covid-19 vaccination was not associated with risk of "any diagnoses" (??), you mean of any covid-19 diagnoses? or any hospital admission??

- **Authors’ response:** We agree this was confusing. We have now corrected to the following:

*“Adolescents vaccinated with at least one dose of a monovalent COVID-19 mRNA vaccine did not experience an increased risk of all-cause hospitalisation as compared to unvaccinated adolescents”*

5. The safety analysis is important.  However, I am surprised the authors did not specifically look at myocarditis/pericarditis since that is where the worry has been, and they even mention these potential adverse events in the research summary of earlier research.

- **Authors’ response:** This comment may perhaps be due to misunderstanding. We have indeed examined myocarditis and pericarditis as specific safety outcomes. These results are presented in Table 2, showing no increased risk of these outcomes in vaccinated individuals.

6. Ethics approval. While informed consent is often waived in register-based studies that is usually not because of their retrospective study design but because of their register-based nature, that already collected data are used, and that the study is simply not feasible/possible if informed consent was required. So the authors may consider removing the argument why informed consent was waived.

- **Authors’ response:** Thank you for noting out this mistake in writing. Corrected.

7. Did really Statistics Sweden identify the exposed? That is usually the job of the National Board of Health and Welfare (or perhaps the Public Health Agency). Matching is however done by Statistics Sweden.

- **Authors’ response:** It is true that the Public Health Agency of Sweden holds the COVID-19 specific data, and that Statistics Sweden does the matching, as we have already written in the paper. However, in order for Statistics Sweden to be able to do the matching they need to retrieve the COVID-19 data from Public Health Agency of, which was the case in the present study.

8. Control selection. there is of course a risk that individuals who chose not to vaccinate themselves did so because they were sure they had had covid-19 (even if not documented), and that would lead to a lower risk of covid-19 among the unexposed (thereby underestimating any protective effect of vaccination) how did the authors tackle this?

- **Authors’ response:** It is correct that undocumented prior infections in the control group may be present, thereby underestimating the protective effect of vaccination. However, it should be noted that our estimates of effectiveness (76%) are very similar to the estimates of 80% from the other studies. Regardless, we have added a sentence in the limitations section of the Discussion, highlighting the problem with undocumented prior infections:

*“Another limitation is that even though we excluded all individuals with documented prior SARS-CoV-2 infection, the estimates of VE could be underestimated if some of the individuals in the unvaccinated control group had acquired immunity from a prior SARS-CoV-2 infection that was either asymptomatic or undocumented.”*

9. I like the dummy analyses with influenza as the outcome. However, I still feel that any vaccination analysis should be adjusted for additional co-variates. I understand that such covariates may not have been available up until "vaccination date" since there is a lag of reporting some data, but perhaps adjust for covariates up until Dec 31, 2021 in your analyses?

- **Authors’ response:** We understand the reviewer’s point. However, the problem with adjusting for a massive number of diagnoses at baseline (apart for the fact that most were uncommon), is the fact that the COVID-19 hospitalizations were uncommon. Thus, the risk of imprecise regression estimates would increase. Perhaps more importantly, baseline characteristics was similar in vaccinated and unvaccinated individuals, as demonstrated in Table 1, except for the variables we actually adjusted for.

10. In the results you state that controls were less often diagnosed with a previous SARS-COV2 infection, but was not such infection an exclusion criteria for control eligibility??

- **Authors’ response:** Yes, prior infection was an exclusion criterion in the cohort estimating VE, but not in the cohort used in the safety analysis.

11. Sensitivity analysis, how can the VE 95%CI for influensa be -51 to 119, if the Vaccine efficiency was -6% (should be in the middle of the 95%CI, and if that is percent, how an it exceed 100%)?

- **Authors’ response:** The reason why the upper end of the 95% CI exceeds 100 is because the VE is based on the odds ratio and the 95% CI surrounding it. VE = 1-odds ratio x 100. We have now revised this section to clarify and improve readability:

  *“The results showed that individuals vaccinated with two doses of COVID-19 vaccine did not experience a lower risk of hospitalisation due to influenza as compared to never vaccinated individuals (VE; -10%, 95% CI [-133, 48], P=0.81), thus indicating an absence of important unmeasured confounding.”*

12. Table 1. the codes for infections by no means cover all infections, only a small proportion.

     a) please clarify that ("selected infections"),

      b) add to the table legends what infections were included.

- **Authors’ response:** We have now changed to “Selected infections”. The infections included are already given in parentheses.

13. F10 is alcohol dependency, it is "acute alcohol intoxication", typically a young person with binge drinking Friday or Saturday night who ends up in the emergency department.

- **Authors’ response:** We are not sure about this comment, and a word might be missing. Regardless, F10 is describing a number of different conditions related to alcohol abuse. The exact meaning depends on the third position in the code, e.g. F100.

14. If you include Crohn's disease (K50) it is strange that you have not included the more common ulcerative colitis (K51)

- **Authors’ response**: Please understand that we only used main diagnoses set during hospital stay, and hospitalizations with a main diagnosis of K50 and K51 was similar in frequency in the study cohort.

15. Minor Table 1. incorrect spelling of "sinusitis"

- **Authors’ response:** Thanks, corrected.
