# Supplementary material for: Safety and effectiveness of monovalent COVID-19 mRNA vaccination and risk factors for hospitalisation caused by the omicron variant in 0.8 million adolescents: A nationwide cohort study in Sweden
Source: PLoS Med. 2023 Feb 21;20(2):e1004127. doi: 10.1371/journal.pmed.1004127 (PMC9990916; doi:10.1371/journal.pmed.1004127)
Supplement: Attachment — Submitted filename: Authors respones 230126.docx [file pmed.1004127.s004.docx]

Requests from Editors:

**GENERAL**

- Thank you for your detailed and considerate responses to previous editor and reviewer comments. Please respond to further outstanding comments as detailed below:
  - **Authors’ response:** Thank you! We have now responded to all remaining comments.

**STATISTICAL REPORTING**

- p values should be reported with a lowercase p. Please amend throughout including tables and figures where relevant
  - **Authors’ response:** Corrected.

**DATA AVAILABILITY STATEMENT**

- Thank you for updating your statement. Please upload this revised statement into the masncuript submission form when you re-submit your manuscript
  - **Authors’ response:** Done.

**AUTHOR SUMMARY**

- What did the researchers do and find – “There were certain strong risk factors….” Suggest instead, “There were specific risk factors for COVID-19 hospitalization including…” What do these findings mean – point 2 starting “In contrast…” – suggest making this a separate bulleted point
  - **Authors’ response:** We are unsure if this comment is a mistake. Under the section “What do these findings mean”, there are 3 bullet points, and the third one starting with “In contrast” is already a separate point. This was raised during the previous round of revision, and corrected then.

**ABSTRACT**

- Methods and findings section - “Strong risk factors for …” suggest alternative to the word strong perhaps “Predominant”
  - **Authors’ response:** Done, changed to “predominant”.

**METHODS**

- Final paragraph – please ensure that (95%) CI has been defined at first use – I couldn’t see that it had but apologies if I have missed it
  - **Authors’ response:** Corrected!

**RESULTS**

- Please report p values using a lowercase p. Please check and amend throughout
  - **Authors’ response:** Done.

**DISCUSSION**

- Page 2, paragraph 1, final line: “… be targeting risk groups…” suggest “at risk” or “high risk” or something similar.
  - **Authors’ response:** Done. The sentence has been changed to the following: *“Taken together, these findings suggest that vaccination of adolescents during the omicron era should primarily be targeting those at high risk of severe disease, such as those with previous infections and development disorders.”*

**SOCIAL MEDIA**

- To help us extend the reach of your research, please provide any Twitter handle(s) that would be appropriate to tag, including your own, your coauthors’, your institution, funder, or lab. Please respond to this email with any handles you wish to be included when we tweet this paper.
  - **Authors’ response:** You may consider the Twitter handles of Uppsala University (@UU_University and @uppsalauni) and Umeå University (@UmeaUniversity and @umeauniversitet).
